# Mechanochemical endovenous Ablation versus RADiOfrequeNcy Ablation in the treatment of primary great saphenous vein incompetence (MARADONA): study protocol for a randomized controlled trial

**DOI:** 10.1186/1745-6215-15-121

**Published:** 2014-04-11

**Authors:** Ramon RJP van Eekeren, Doeke Boersma, Suzanne Holewijn, Anco Vahl, Jean Paul PM de Vries, Clark J Zeebregts, Michel MPJ Reijnen

**Affiliations:** 1 Department of Surgery, Rijnstate Hospital, Wagnerlaan 55, Arnhem 6815 AD, The Netherlands; 2 Department of Vascular Surgery, St Antonius Hospital, Koekoekslaan 1, Nieuwegein 3435 CM, The Netherlands; 3 Department of Surgery, Onze Lieve Vrouwe Gasthuis, Oosterpark 9, Amsterdam 1091 AC, The Netherlands; 4 Department of Surgery, BovenIJ Hospital, Statenjachtstraat 1, Amsterdam 1034 CS, The Netherlands; 5 Department of Vascular Surgery, University Medical Centre Groningen, University of Groningen, Hanzeplein 1, Groningen 9700 RB, The Netherlands

**Keywords:** Mechanochemical ablation, ClariVein, Radiofrequency ablation, Varicose vein, Therapy, Treatment, MOCA, Outcome

## Abstract

**Background:**

Radiofrequency ablation (RFA) is associated with an excellent outcome in the treatment of great saphenous vein (GSV) incompetence. The use of thermal energy as a treatment source requires the instillation of tumescence anesthesia. Mechanochemical endovenous ablation (MOCA) combines mechanical endothelial damage, using a rotating wire, with the infusion of a liquid sclerosant. Tumescence anesthesia is not required. Preliminary experiences with MOCA showed good results and low post-procedural pain.

**Methods/Design:**

The MARADONA (Mechanochemical endovenous Ablation versus RADiOfrequeNcy Ablation) trial is a multicenter randomized controlled trial in which 460 patients will be randomly allocated to MOCA or RFA. All patients with primary GSV incompetence who meet the eligibility criteria will be invited to participate in this trial. The primary endpoints are anatomic and clinical success at a one-year follow-up, and post-procedural pain. The secondary endpoints are technical success, complications, operation time, procedural pain, disease-specific quality of life, time taken to return to daily activities and/or work, and cost-efficiency analyses after RFA or MOCA. Both groups will be evaluated on an intention to treat base.

**Discussion:**

The MARADONA trial is designed to show equal results in anatomic and clinical success after one year, comparing MOCA with RFA. In our hypothesis MOCA has an equal anatomic and clinical success compared with RFA, with less post-procedural pain.

**Trial registration:**

Clinicaltrials NCT01936168

## Background

Varicose veins are a common problem in the Western world. Epidemiological studies show that 21% of adults have some form of varicose veins [1-3], with women being more affected than men [3,4]. The incidence of varicose veins increases steadily with age and is among the top ten complaints for which people visit their general practitioner. The main risk factors include: prolonged standing or sitting, pregnancy, sex and age [5]. The symptoms of varicose veins range from cosmetic complaints to venous ulcers [6].

High ligation and stripping of the great saphenous vein (GSV) has been the gold standard for GSV incompetence for more than 100 years [7]. Surgery is performed under general or spinal anesthesia and is related to a high recurrence rate of 18 to 40% after five years [8,9]. In addition, surgery may lead to significant postoperative symptoms (particularly pain and hematoma) and carries a risk of injury to the saphenous nerve [10,11].

Endovenous techniques have been developed for the treatment of varicose veins [12]. Endovenous laser ablation (EVLA) and radiofrequency ablation (RFA) are now widely accepted techniques and are frequently used in practice [13,14]. They are related to less hematoma, pain, and superior cosmetics and earlier resumption of normal activities and work when compared to traditional surgical stripping [15,16]. Thermal ablative modalities, however, carry the risk of damaging the surrounding tissues of the vein. For this reason, patients are treated with tumescence anesthesia, which requires multiple punctures around the vein. Despite the use of tumescence anesthesia there are still a subset of patients who have postoperative pain, which can last for weeks [17,18].

Mechanochemical endovenous ablation (MOCA), using the ClariVein® device (Vascular Insights, Madison, CT, United States), uses a rotating wire in a catheter to create mechanical damage to the endothelium of the vessel. At the same time, a sclerosant is infused at the end of the catheter, causing chemical damage to the vein wall. With MOCA, the vein wall is not heated and tumescence anesthesia is redundant. Subsequently complications that occur in thermal ablative modalities such as pain, hematoma, induration and nerve injury could be reduced.

The safety and efficacy of MOCA was shown in the first human study [19]. In this study, 30 patients with primary GSV insufficiency were treated using sodium tetradecyl sulfate (Sotradecol). At six months the anatomical success was 97% [19]. After a follow-up period of two years, 27 of the 28 (anatomical success 96%) treated GSV were occluded [20]. Several reports have confirmed the efficacy of MOCA, with occlusion rates varying from 94 to 97% [21-23]. No major complications such as deep vein thrombosis, pulmonary embolism or nerve injury were observed in all previous studies. Moreover, MOCA was associated with lower post-procedural pain and faster recovery than RFA [24]. To date, no studies have been performed to compare MOCA with other endovenous techniques in the treatment of varicose veins. The current study has been designed to compare MOCA for the treatment of GSV insufficiency to RFA in a multicenter randomized controlled trial.

## Methods/Design

### Study design

A prospective multicenter randomized clinical trial was designed to compare RFA and MOCA in the treatment of GSV incompetence. Patients will be included at the outpatient departments of the participating hospitals. The procedures are performed or supervised by dedicated vascular surgeons, who have performed more than 20 procedures of both treatment modalities.

### Study objectives

The aim of the study is to show that MOCA is associated with less post-procedural pain than RFA, with comparable anatomical and clinical success rates.

### Sample size calculation

The assumption has been made that MOCA will have a similar anatomical and clinical success rate at one year when compared to RFA. For a non-inferiority trial with an effect size (anatomical success) of 93% and a margin of 7%, 210 patients per group are needed (alpha 5%, power 80%). The effect size of 93% refers to an estimated anatomical success rate at a one year follow-up period for both groups. Taking into account 10% dropouts in each arm, 230 patients need to be included in each study arm.

A sample size calculation will also be performed for the second primary endpoint on the hypothesis that MOCA will have lower post-procedural pain, as measured by a 100-point VAS (Visual Analog Scale) score during the first two weeks after surgery. To evaluate a 30% reduction in post-procedural pain, 58 patients per group are needed (alpha 5%, power 80%). This analysis will be performed after the inclusion and randomization of at least all 58 patients in each group.

### Setting

Patients will be recruited from the following Dutch centers: Rijnstate Hospital, Arnhem; Antonius Hospital, Nieuwegein; Onze Lieve Vrouwe Gasthuis, Amsterdam; BovenIJ Hospital, Amsterdam; University Medical Center Groningen, Groningen, The Netherlands. In all of the participating centers, each surgeon performing the procedures must have treated at least 20 patients with each technique prior to treating patients who participate in the MARADONA trial to prevent a learning curve bias.

The total study duration will be seven years: the recruitment period will take two years and thereafter patients will be evaluated for a period of five years post-procedure.

### Primary endpoints

The primary endpoints are anatomic and clinical success and post-procedural pain after treatment for GSV incompetence with RFA or MOCA. Anatomic success will be measured one year after treatment using duplex ultrasonography. Clinical success will be measured using the Venous Clinical Severity Score (VCSS). Post-procedural pain will be evaluated using a 100-point VAS during the first two weeks after the treatment.

### Secondary endpoints

The secondary endpoints are technical success, complications, operation time, pain during treatment, disease-specific quality of life, time to return to daily activities and/or work, and cost-efficiency analyses after RFA or MOCA.

### Ethical considerations

A patient who meets the entry criteria is fully informed about the trial and provided with a patient information and consent form. Patients willing to participate in the study are included after signing the informed consent form. This study is conducted in accordance with the principles of the Declaration of Helsinki and Good Clinical Practice guidelines. The study is approved by the Medical Ethics committee of Nijmegen (CMO 2011/091) and the local institutional board of each participating center.

### Safety and quality control

#### Data safety monitoring board

The Data Safety Monitoring Board (DSMB) will review safety and make recommendations regarding the conduct of the study to the steering committee, and to the accredited Medical Ethical Board (METC) that approved the study protocol. An interim safety analysis will be performed at six-months after initiation of the trail. This analysis will include at least 75 patients.

#### Adverse and severe adverse events

Adverse events (AE) are defined as any undesirable experience occurring to a participant during the study, whether considered related to the investigational device or not. This definition includes events occurring during a hospital stay of up to 30 days of follow-up. Any underlying disease that was present at the time of enrollment is not reported as an AE, but any increase in the severity of the underlying disease will be reported as an AE. All AEs will be monitored from the time of enrolment through the 30-day follow-up visit. AEs will be recorded on the case record forms. A description of the event, including the start date, end date, action taken, and the outcome will be provided.

A severe adverse event is any event leading to death, deep venous thrombosis, and neurological complications.

Data on AEs will be reported to the DSMB and to the accredited METC via 'Toetsingonline’ on the website of the Central Committee on Research involving Human Subjects (CCMO).

### Inclusion criteria

The inclusion criteria are: primary GSV incompetence; C2 to C5 varicose veins; diameter of the GSV at the saphenofemoral junction ≥ 3 or ≤ 12 millimeter; aged between 18 and 80 years; andwritten informed consent.

### Exclusion criteria

The exclusion criteria are: C6 varicose veins; previous surgery or treatment for varicose veins (such as a crossectomy, surgical strip, or thermal ablative procedure); oral anticoagulant; pregnancy or lactation; previous deep venous thrombosis; immobilization; contraindication or known allergy for sclerosant; coagulation disorders or an increased risk of thromboembolism; severe renal insufficiency; and/or severe liver insufficiency.

### Recruitment

**Figure 1:**
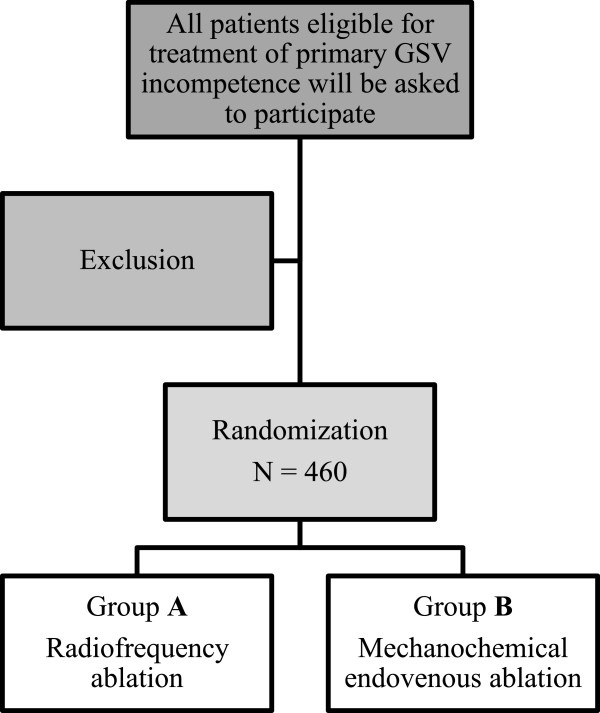
**This figure illustrates the study design.** A total of 460 patients will be randomized to radiofrequency ablation (N = 230) or mechanochemical endovenous ablation (N = 230). GSV, great saphenous vein.

A total of 460 patients with primary great saphenous incompetence are to be included in the MARADONA trial after signing informed consent (Figure 1). The pre-procedural status will be determined by the Clinical Etiology Anatomy Pathophysiology (CEAP) score [25] and VCSS [26].

After randomization, the GSV is obliterated in day treatment: study arm 1: (RFA); study arm 2: (MOCA).

### Randomization

Randomization will be performed during the outpatient department visit in which the patient is included. Patients will be randomized by the responsible surgeon to one of the treatment arms, using a web-based randomization tool (Research Manager, NOVA Business Software, Zwolle, The Netherlands). Randomization will be performed by blocks with stratification for participating centers.

### Treatment details

#### Radiofrequency ablation (RFA)

During RFA, radiofrequent energy is used to heat the vein wall of the GSV. The catheter is inserted into the vein and direct energy is delivered to the endothelium with the result of collapsing and sealing the vein. During the MARADONA study, all participating centers are using the VNUS ClosureFAST™ catheter (VNUS Medical Technologies, San Jose, California, United States). The VNUS ClosureFAST™ catheter contains a 7 cm long heating element at the end of the catheter, used for 'segmental’ ablation. A temperature of 120°C is generated in the heating element during a 20 second treatment cycle.

The insufficient GSV is punctured, ultrasound-guided, and a guidewire inserted. An introducer sheath is placed over the guidewire. The catheter is then introduced and positioned 2 cm distal to the saphenofemoral junction using ultrasound guidance. The tumescence anesthesia is delivered along the entire segment to be treated. An approximate volume of 10 mL per centimeter-treated vein is used. The patient is placed in a horizontal position and the catheter is activated. Every 20 seconds a new 7 cm segment of the GSV is treated after withdrawal. The most proximal segment of the GSV is treated with two cycles as recommended by the manufacturer. This process is repeated until the entire incompetent vein is ablated. After treatment, the deep venous system is controlled by ultrasound.

A compression stocking (20 to 30 mmHg) will be applied continuously for 24 hours and then daily for two weeks. Patients may resume normal activities immediately after the procedure. No concomitant phlebectomies or additional sclerotherapy is performed.

#### Mechanochemical endovenous ablation (MOCA)

During MOCA, the ClariVein® device is used to induce a combination of mechanical and chemical damage to the vein wall. The ClariVein® device is an infusion catheter which is designed to administer a sclerosant in the incompetent vein through an opening at the end of the catheter. An iron wire extends through the whole catheter, with a small iron ball at the end. The purpose of the rotating wirer is fourfold: (1) promoting the coagulation activation by minimal mechanical damage to the endothelium, (2) inducing a vasospasm which reduces the diameter of the vein, (3) increasing the action of sclerosant by an increase in surface, (4) ensuring an even distribution of the sclerosant at the endothelium. The catheter together with the iron wire is connected to a motorized handle that allows for the rotation of the metal wire.

The insufficient GSV is punctured, ultrasound-guided and a guidewire inserted. A 4 Fr introduction sheath is introduced and subsequently the ClariVein® catheter is inserted and the tip of the iron wire is placed near the saphenofemoral junction, 0.5 cm below the superficial epigastric vein, as verified by ultrasound. The patient is treated in a horizontal position. The wire is then activated for 10 seconds in order to induce vasospasm. The device is slowly withdrawn with a speed of about 7 seconds per centimeter, while the sclerosant is continuously injected using 2 mL 3% polidocanol for the first 10 to 15 cm and 1.5% polidocanol for the remainder of the GSV. After treatment, the deep venous system is controlled with ultrasound. A compression stocking (20 to 30 mmHg) will be applied continuously for 24 hours and then daily for two weeks. Patients may resume normal activities immediately after the procedure. No concomitant phlebectomies or additional sclerotherapy is performed.

Polidocanol (Aethoxysklerol) is used as sclerosant, which is the only registered sclerosant in the Netherlands and is regularly used for sclerotherapy of reticular veins. The maximum amount of polidocanol depends on the weight of the patient, and will always be less than 2 mg/kg. Prior to treatment, the maximum allowable amount of polidocanol is determined for each patient. Then the length of the treated vein is measured. Both values are listed on the case report forms.

### Follow-up treatment periods

After four weeks, one, two and five years, patients are seen at the outpatient clinic to observe anatomical and clinical success (Table 1). Ultrasound duplex imaging, VCSS, CEAP score and quality of life scores are measured at all above mentioned time points. Ultrasound duplex imaging is done according to a standardized protocol for all participating hospitals.

**Table 1:** Flow chart of the trial

	**Study period**					
	**Screening**	**Procedure**	**4 weeks**	**1 year**	**2 years**	**5 years**
**Outpatient visit**	X		X	X	X	X
**Physical examination**	X		X	X	X	X
**Informed consent**	X					
**Inclusion criteria**	X					
**Randomization**		X				
**CEAP/VCSS**	X		X	X	X	X
**Ultrasound**	X		X	X	X	X
**Pain score**		X				
**AVVQ**	X		X	X	X	X
**SF-36**	X		X	X	X	X

AVVQ: Aberdeen Varicose Vein Questionnaire; CEAP: Clinical Etiology Anatomy Pathophysiology classification; SF-36: Short Form 36-Item Health Survey; VCSS: Venous Clinical Severity Score.

Post-procedural pain is evaluated using a linear VAS score of 0–100 mm during 2 weeks after treatment. After the 4-week control any small branch varicosities may be treated when indicated.

### Quality of life scores

The Short Form-36 (SF-36) is a multidimensional measurement of general health. It yields eight domains of functional health and well-being scores. The 'Dutch translated’ Aberdeen Varicose Vein Questionnaire [27] (AVVQ) is a validated disease-specific quality of life measurement for chronic venous insufficiency.

Both questionnaires are completed preoperatively, after 4 weeks, one, two, and five years of follow-up.

### Data collection

Data will be collected at the recruitment centre by means of case report forms. Copies of the case report forms will be sent to the coordinating center (Rijnstate Hospital, Arnhem, The Netherlands) where all data will be entered in a validated data management system (Research Manager, NOVA Business Software, Zwolle, The Netherlands) and controlled by an independent monitor. The participating centers will be informed about the current status of recruitment and adverse events via a newsletter every three months. Additionally, there will be regular contact between the principal investigator and the contact persons from the participating centers.

### Statistical analyses

The study results will be evaluated based on an intention-to-treat analysis. Data concerning the one, two and five year follow-up will be analyzed for both study groups on an intention-to-treat manner by student t-test (normal distribution) of Mann Whitney U-test (skewed distribution). Obliteration rates will be presented as Kaplan Meier curves including censoring.

### Publication of data

Data will be published after a follow-up period of one, two and five years, regardless of the outcome of the study, under the responsibility of MMPJ Reijnen, MD, PhD. Co-authorship will be assigned according to the 'Uniform Requirements for Manuscripts Submitted to Biomedical Journals: Writing and Editing for Biomedical Publication' of the International Committee of Medical Journal Editors.

### Definitions of terms used for the study

Anatomical success: occlusion of the treated GSV segment, measured with duplex.

Clinical success: objective improvement of clinical outcome after treatment, measured with the Venous Clinical Severity Score (VSCC) of at least 1.

Technical success: initial technical success rate of the procedure, where the catheter can be safely placed at a defined distance from the saphenofemoral junction and the GSV can be treated without technical problems.

Failure of treatment: type 1 (non-occlusion) - the treated vein failed to occlude initially and never occluded during the follow-up; type 2 (recanalization) - the treated vein occluded directly after treatment, but recanalized, partly (>10 cm) or completely, at a later time point during follow-up; type 2a - recanalization of the entire treated segment of the vein; type 2b - partial recanalization (open segment > 10 cm).

Post-procedural complications: complications occurring within 30 days after treatment. Major complications to include deep venous thrombosis, pulmonary embolism, skin burn, saphenous neuralgia. Minor complications to include ecchymosis, superficial phlebitis, hyperpigmentation, induration, wound infection of the puncture site, prolonged pain > one week. Sclerosans-related complications.

Operation time: time of the procedure, starting from puncture of the vein to extracting of the catheter, skin-to-skin contact.

## Discussion

Since the introduction of minimal invasive modalities for the treatment of great saphenous incompetence, many new techniques have been developed and introduced to the market. While most techniques constantly report anatomical success rates over 90% [13-16], more emphasis is on secondary treatment outcomes such as post-procedural pain, hematoma, quality of life and returning to normal activities. Therefore, the optimal treatment for GSV incompetence is unclear.

The aim of the present randomized trial is two-fold. First, it aims to compare anatomical and clinical success at one year after MOCA, with RFA. Several randomized trials have compared RFA with conventional surgery, endovenous laser ablation and foam sclerosis [12]. These studies showed the superiority of EVLA in terms of anatomical success at one to five years after surgery, although newer radiofrequency devices have similar results [13,28].

Second, it aims to demonstrate that MOCA is associated with a significant reduction in post-procedural pain after treatment. Pain after endothermal ablation is considerable and probably an underreported complication in the literature. Recent studies have shown less post-procedural pain after RFA comparing with EVLA [17,29]. Therefore, accompanied with similar occlusion rates with the newer RFA devices, we have assumed RFA to be one of the 'gold standard’ techniques in minimal invasive treatment for GSV incompetence. In order to have sufficient power, the trial was designed using a non-inferiority principle.

In conclusion, the MARADONA trial is multicenter randomized controlled trial that aims for a reduction in post-procedural pain after MOCA compared with RFA, with a similar anatomical and clinical success.

## Trial status

The MARADONA trial started with inclusion of patients in July 2013. In December 2013, 50 patients were randomized after signing informed consent.

## Abbreviations

AE: adverse event; AVVQ: Aberdeen Varicose Vein Questionnaire; CEAP: clinical etiology anatomy pathophysiology; DSMB: data safety monitoring board; EVLA: endovenous laser ablation; GSV: great saphenous vein; MOCA: mechanochemical endovenous ablation; SF-36: Short Form-36; VAS: visual analog scale; VCSS: Venous Clinical Severity Score.

## Competing interests

The authors declare that they have no competing interests.

## Authors’ contributions

RVE drafted the manuscript, initiated and designed the study, performs data acquisition and revised the manuscript. DB co-authored the writing of the manuscript, initiated and designed the study, and revised the manuscript. SH was the coordinating trial investigator, participated in the trial design and sample size calculation, performs data management and coordination between study centers, and revised the manuscript. AV contributed to the scientific accuracy of the manuscript, performs data acquisition, and revised the manuscript. JPV initiated and designed the study, performs data acquisition, and revised the manuscript. CZ contributed to the scientific accuracy of the manuscript, performs data acquisition, and revised the manuscript. MR responsible trial coordinator, initiated and designed the study, performs data acquisition and coordination between study centers, and revised the manuscript. All authors read and approved the final manuscript.
